# Supplementary material for: High-fat diet fuels prostate cancer progression by rewiring the metabolome and amplifying the MYC program
Source: Nat Commun. 2019 Sep 25;10:4358. doi: 10.1038/s41467-019-12298-z (PMC6761092; doi:10.1038/s41467-019-12298-z)
Supplement: Supplementary file 1 — Supplementary Information [file 41467_2019_12298_MOESM1_ESM.pdf]

Supplementary Information

High-fat diet fuels prostate cancer progression by rewiring the metabolome and amplifying the MYC program

Labbé *et al.*

Supplementary Figures

Supplementary Figure 1: High-fat diet and MYC over expression alters the prostatic metabolome

Supplementary Figure 2: High-fat diet and MYC over expression alters the global prostatic chromatin profile and transcriptome

Supplementary Figure 3: The MYC transcriptional signature is amplified by high-fat diet

Supplementary Figure 4: The saturated fat-induced MYC signature does not correlate with BMI

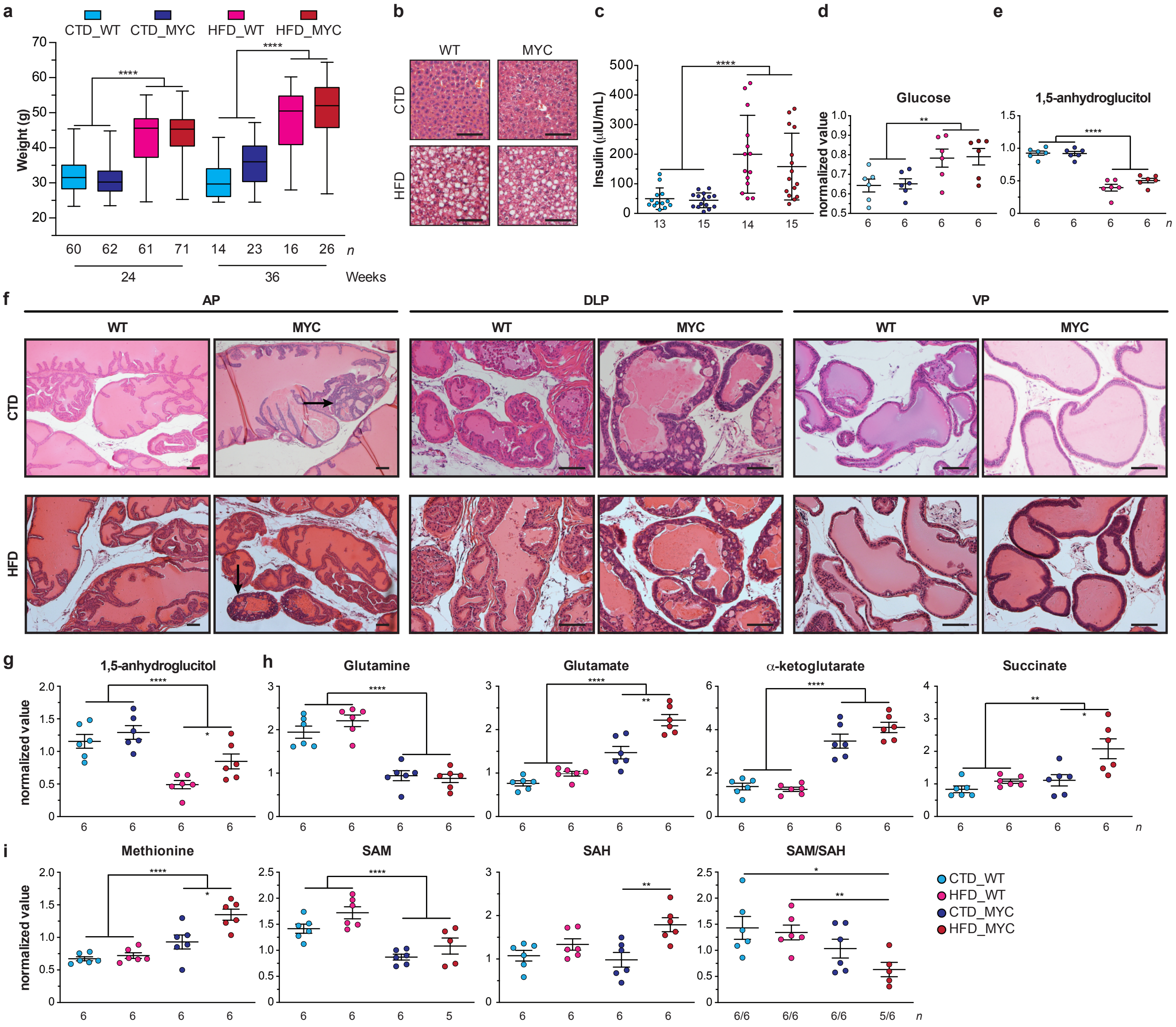

Supplementary Figure 1: High-fat diet and MYC over expression alters the prostatic metabolome. (**a**) Mice fed a high fat diet maintain an increased body weight at 24 and 36 weeks of age (*n* = biologically independent animals; two-way ANOVA, median, whiskers ± min/max; *****P*<0.0001). (**b, c, d, e**) HFD results in liver steatosis (**b**, hematoxylin and eosin (H&E) staining; scale bar = 100 μM), increased circulating insulin (**c**, *n* = biologically independent animals; two-way ANOVA, mean ± s.d.; three samples were removed from the analysis - see methods; *****P*<0.0001) and glucose (**d**, *n* = biologically independent animals; two-way ANOVA, mean ± s.e.m.; ***P*<0.01; obtained from metabolic profiling), and decreased circulating 1,5-anhydroglucitol (**e**, *n* = biologically independent animals; two-way ANOVA, mean ± s.e.m.; *****P*<0.0001; obtained from metabolic profiling) in 12-week-old mice. (**f**) Representative H&E staining of prostate lobes from 12-week-old mice fed a CTD diet depicting representative normal (WT) and transformed (MYC; prostatic intraepithelial neoplasia (PIN) in the AP (*arrow*), DLP and VP; scale bar = 100 μM) prostatic glands. (**g, h, i**) Quantification of metabolites related to the HFD-associated phenotype (**g**), demethylation (**h**) and methylation processes (**i**) in the VP of 12-weeks-old mice (*n* = biologically independent VP; mean ± s.e.m.; HFD vs. CTD or MYC vs. WT two group comparisons: two-way ANOVA; single group comparisons: unpaired *t* test, ; **P*<0.05, ***P*<0.01, ****P*<0.001, *****P*<0.0001). Complete details on changes in prostatic and serum metabolites are shown in Supplementary Data 2 and 15, respectively. Source data are provided as a Source Data file.

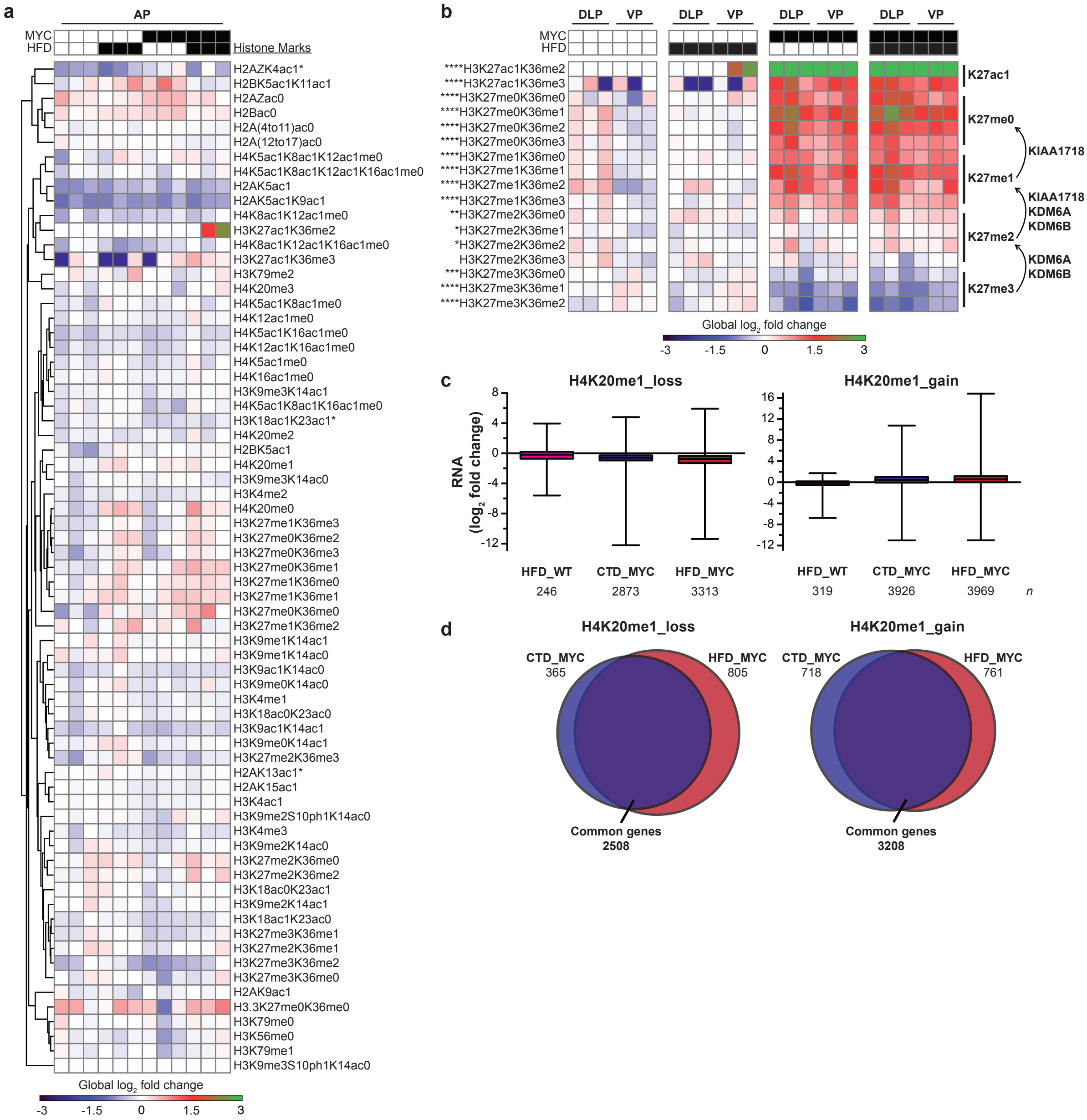

Supplementary Figure 2: High-fat diet and MYC over expression alters the global prostatic chromatin profile and transcriptome. (**a**) Global chromatin profiling of AP lobes (histone marks levels relative to the DLP, VP and AP CTD_WT median values; MYC vs. WT comparisons, unpaired *t* test; *P<0.05; Supplementary Data 6). (**b**) MYC overexpression, irrespective of diet, results in a significant hypomethylation of the H3K27 mark (MYC vs. WT comparisons, unpaired *t* test; *P<0.05, **P<0.01, ****P<0.0001). (**c**) Decrease in H4K20me1 gene body-associated mark (relative to the CTD_WT condition) is associated with a lower level of gene transcription (*left*) while its gain to higher gene transcription (*right*) especially in the context of MYC overexpression (median, whiskers ± min/max). (**d**) Venn diagram showing overlapping genes between the CTD_MYC and HFD_MYC conditions that demonstrate either a decrease (*left*) or an increase (*right*) in H4K20me1 gene body-associated mark relative to the CTD_WT condition.

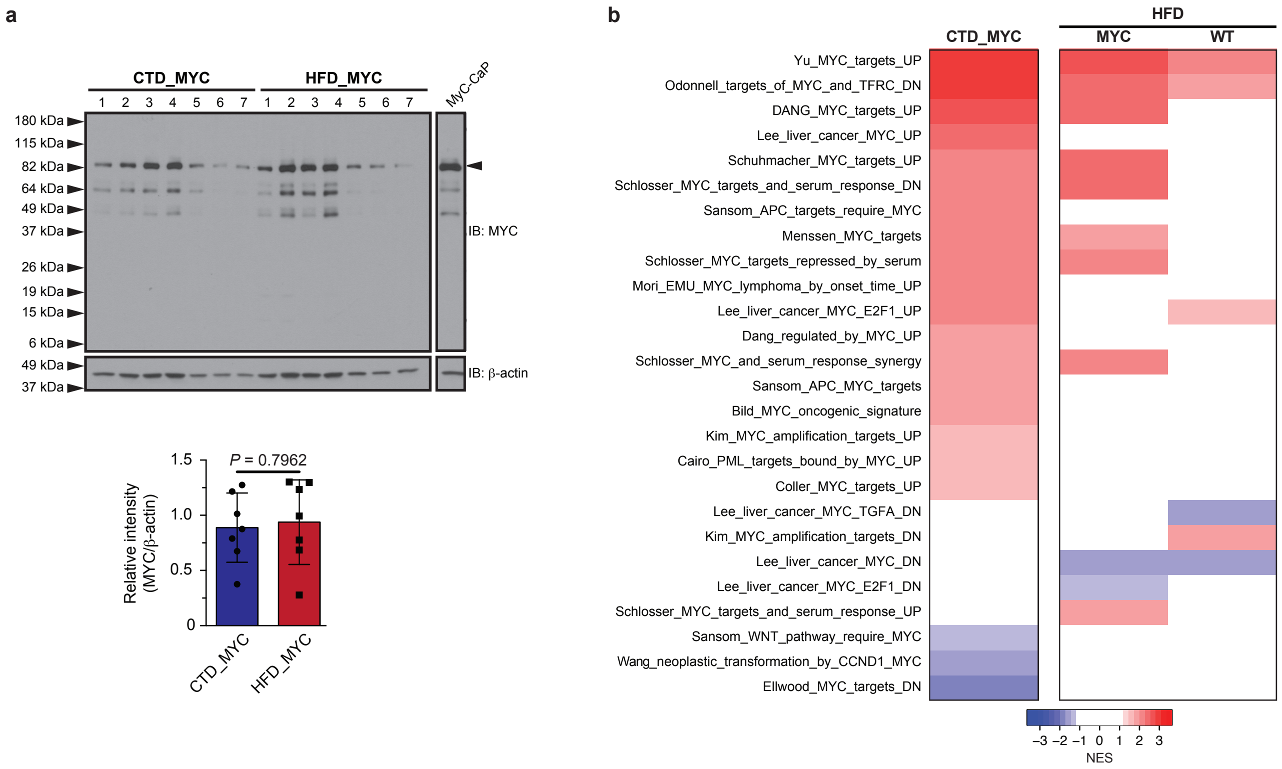

Supplementary Figure 3: The MYC transcriptional signature is amplified by high-fat diet. (**a**) MYC transgene protein level (VP, *n* = 7 biologically independent lobes) and its quantification (unpaired *t* test, mean ± s.d.). MyC-CaP cell lysates were used as positive controls for MYC transgene expression (*arrow*). (**b**) Heatmap representation of all MYC-related gene sets, enriched (*red*) or depleted (*blue*) either by MYC overexpression (*left column*) or HFD in a MYC or WT context (GSEA, Chemical and Genetic Perturbation). Leading edge genes included in the enriched gene sets, together with both MYC-related Hallmark gene sets (*n* = 610 genes) were used to derive the murine prostatic MYC signature (Supplementary Data 9). Source data are provided as a Source Data file.

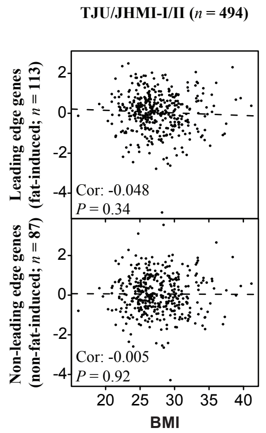

Supplementary Figure 4: The saturated fat-induced MYC signature does not correlate with BMI as observed in the TJU/JHMI-I/II cohorts ($n = 494$).

Supplementary Tables

Supplementary Table 1: Murine diets – Selected nutrients

Supplementary Table 2: MYC signature score based on randomly picked MYC_targets_V1 genes in relation to the risk of prostate cancer death among men diagnosed with non-metastatic prostate cancer

Supplementary Table 3: Univariate survival analysis – Validation cohorts

Supplementary Table 4: Multivariate survival analysis including gleason grade – Validation cohorts

Supplementary Table 5: Multivariate survival analysis including Cell Cycle Progression score – Validation cohorts

Supplementary Table 6: Murine diets – Formula

Supplementary Table 7: Metabolomics – Internal standards

Supplementary Table 8: Metabolomics – Elution gradient profiles

Supplementary Table 9: Metabolomics – MS details

Supplementary Table 1: Murine diets – Selected nutrients

	Control diet TD.130838		High fat diet TD.06414	
**Nutrient**	**% by weigh**	**% kcal from**	**% by weigh**	**% kcal from**
Protein	16.5	18.4	23.5	18.4
Carbohydrate	63.9	71.1	27.3	21.3
Fat	4.2	10.5	34.3	60.3
Soybean Oil	*2*	*5*	*3*	*5.3*
Lard	*2*	*5*	*31*	*54.7*
kcal/g	3.6		5.1	

Supplementary Table 2: MYC signature score based on randomly picked MYC_targets_V1 genes in relation to the risk of prostate cancer death among men diagnosed with non-metastatic prostate cancer

MYC score	N	OR (95% CI)*	OR (95% CI)‡	OR (95% CI)§
Tertile 1 (low)	17	1.00	1.00	1.00
Tertile 2	19	1.17 (0.57, 2.44)	1.03 (0.47, 2.30)	0.96 (0.43, 2.16)
Tertile 3 (high)	26	1.79 (0.90, 3.64)	1.71 (0.81, 3.70)	1.66 (0.78, 3.61)
P, linear trend†		0.094	0.152	0.170

N = lethal events; OR = odds ratio; CI = confidence interval.

*Logistic regression model adjusted for age and year at diagnosis (continuous).

†Estimated by modeling tertiles of MYC score as continuous variable (tertile 1=0, tertile 2=1, tertile 3=2).

‡Logistic regression model adjusted for age and year at diagnosis (continuous), and Gleason grade (continuous: <7, 3+4, 4+3, >7).

§Logistic regression model adjusted for age and year at diagnosis (continuous), Gleason grade (continuous: <7, 3+4, 4+3, >7), and BMI at diagnosis (continuous).

Supplementary Table 3: Univariate survival analysis – Validation cohorts

Biochemical recurrence				
**Variable**	**HR**	**2.50%**	**97.50%**	***P***
SFI-induced MYC signature	1.09	0.95	1.24	0.212
log2(PSA)	1.15	1.03	1.28	0.016
Seminal vesicle invasion	2.06	1.60	2.65	<0.001
Surgical margins	1.42	1.08	1.88	0.014
Extracapsular extension	1.55	1.21	1.97	<0.001
Lymph node invasion	1.98	1.32	2.97	<0.001
Cell Cycle Progression score	1.11	0.97	1.27	0.139
Gleason grade	1.67	1.33	2.11	<0.001
**Metastatic progression**				
**Variable**	**HR**	**2.50%**	**97.50%**	***P***
SFI-induced MYC signature	1.61	1.18	2.19	0.003
log2(PSA)	1.15	0.91	1.46	0.240
Seminal vesicle invasion	3.43	2.12	5.53	<0.001
Surgical margins	1.90	1.12	3.23	0.017
Extracapsular extension	3.15	1.74	5.70	<0.001
Lymph node invasion	4.60	2.67	7.91	<0.001
Cell Cycle Progression score	1.64	1.35	1.99	<0.001
Gleason grade	5.37	2.88	10.04	<0.001
**Prostate cancer specific mortality**				
**Variable**	**HR**	**2.50%**	**97.50%**	***P***
SFI-induced MYC signature	1.53	0.95	2.45	0.079
log2(PSA)	1.39	0.99	1.97	0.058
Seminal vesicle invasion	4.49	2.16	9.35	<0.001
Surgical margins	1.60	0.74	3.47	0.229
Extracapsular extension	2.31	1.03	5.16	0.041*
Lymph node invasion	4.83	2.23	10.48	<0.001
Cell Cycle Progression score	1.64	1.24	2.17	<0.001
Gleason grade	4.48	1.82	11.03	0.001

*Gleason grade groups 3, 4, 5 (ref = groups 1 and 2)

Supplementary Table 4: Multivariate survival analysis including gleason grade – Validation cohorts

**Biochemical recurrence**				
**Variable**	**HR**	**2.50%**	**97.50%**	***P***
SFI-induced MYC signature	1.04	0.91	1.19	0.555
log2(PSA)	1.09	0.97	1.22	0.168
Seminal vesicle invasion	1.71	1.29	2.28	<0.001
Surgical margins	1.46	1.09	1.94	0.010
Extracapsular extension	1.16	0.88	1.53	0.291
Lymph node invasion	1.73	1.13	2.63	0.011
Gleason grade	1.49	1.16	1.90	0.001
**Metastatic progression**				
**Variable**	**HR**	**2.50%**	**97.50%**	***P***
SFI-induced MYC signature	1.45	1.06	1.97	0.020
log2(PSA)	1.01	0.8	1.29	0.905
Seminal vesicle invasion	1.87	1.11	3.17	0.020
Surgical margins	1.68	0.99	2.84	0.055
Extracapsular extension	1.62	0.84	3.11	0.149
Lymph node invasion	2.75	1.54	4.92	<0.001
Gleason grade	3.84	2.01	7.33	<0.001
**Prostate cancer specific mortality**				
**Variable**	**HR**	**2.50%**	**97.50%**	***P***
SFI-induced MYC signature	1.32	0.85	2.05	0.212
log2(PSA)	1.29	0.92	1.79	0.138
Seminal vesicle invasion	2.86	1.24	6.58	0.014
Surgical margins	1.45	0.68	3.09	0.330
Extracapsular extension	0.88	0.35	2.17	0.777
Lymph node invasion	2.51	1.07	5.89	0.034
Gleason grade	3.37	1.31	8.65	0.012

*Gleason grade groups 3, 4, 5 (ref = groups 1 and 2)

Supplementary Table 5: Multivariate survival analysis including Cell Cycle Progression score – Validation cohorts

Biochemical recurrence				
**Variable**	**HR**	**2.50%**	**97.50%**	***P***
SFI-induced MYC signature	1.05	0.92	1.20	0.464
log2(PSA)	1.08	0.97	1.21	0.177
Seminal vesicle invasion	1.88	1.42	2.49	<0.001
Surgical margins	1.45	1.09	1.92	0.011
Extracapsular extension	1.18	0.90	1.55	0.241
Lymph node invasion	1.87	1.23	2.85	0.003
Cell Cycle Progression score	1.12	0.97	1.30	0.113
**Metastatic progression**				
**Variable**	**HR**	**2.50%**	**97.50%**	***P***
SFI-induced MYC signature	1.36	1.03	1.80	0.032
log2(PSA)	0.95	0.75	1.19	0.645
Seminal vesicle invasion	2.68	1.58	4.54	<0.001
Surgical margins	1.70	1.01	2.86	0.046
Extracapsular extension	1.64	0.85	3.14	0.138
Lymph node invasion	3.31	1.86	5.89	<0.001
Cell Cycle Progression score	1.69	1.37	2.09	<0.001
**Prostate cancer specific mortality**				
**Variable**	**HR**	**2.50%**	**97.50%**	***P***
SFI-induced MYC signature	1.35	0.90	2.00	0.142
log2(PSA)	1.15	0.83	1.60	0.393
Seminal vesicle invasion	3.83	1.68	8.74	0.001
Surgical margins	1.49	0.69	3.20	0.311
Extracapsular extension	0.90	0.37	2.19	0.823
Lymph node invasion	2.95	1.30	6.70	0.010
Cell Cycle Progression score	1.66	1.25	2.21	<0.001

Supplementary Table 6: Murine diets – Formula

	Control diet		High fat diet	
	TD.130838		TD.06414	
**Ingredient**	**g/kg**	**g/3.6 (Kcal/g)**	**g/kg**	**g/5.1 (Kcal/g)**
Casein	186	51.7	265	52.0
Maltodextrin	112.5	31.3	160	31.4
L-Cystine	2.8	0.8	4	0.8
Sucrose	63.2	17.6	90	17.6
Cellulose	46	12.8	65.5	12.8
Soybean Oil	20	5.6	30	5.9
**Lard**	**20**	**5.6**	**310**	**60.8**
**Corn Starch**	**496.25**	**137.8**	**NA**	**NA**
Calcium Phosphate, dibasic	2	0.6	3.4	0.7
Choline Bitartrate	2.15	0.6	3	0.6
Mineral Mix*	34	9.4	48	9.4
Vitamin Mix†	15	4.2	21	4.1
Red Food Color	0.1	0.0	NA	NA
Blue Food Color	NA	NA	0.1	0.0

*AIN-93G-MX (94046)
†AIN-93-VX (94047)

Supplementary Table 7: Metabolomics – Internal standards

Method	Internal Standard
LC Neg	D7-glucose, d3-methionine, d3-leucine, d8-phenylalanine, d5-tryptophan, bromophenylalanine, d15-octanoic acid, d19-decanoic acid, d27-tetradecanoic acid, d35-octadecanoic acid, d2-eicosanoic acid
LC HILIC	D35-octadecanoic acid, d5-indole acetic acid, bromophenylalanine, d5-tryptophan, d4-tyrosine, d3-serine, d3-aspartic acid, d7-ornithine, d4-lysine
LC Pos	D7-glucose, d3-methionine, d3-leucine, d8-phenylalanine, d5-tryptophan, bromophenylalanine, d4-tyrosine, d5-indole acetic acid, d5-hippuric acid, amitriptyline, d9-progesterone, d4-dioctylphthalate

LC = Liquid Chromatography
HILIC = Hydrophilic Interaction Liquid Chromatography

Supplementary Table 8: Metabolomics – Elution gradient profiles

Reverse Phase Pos and Neg Gradient Profile			
**Time (min)**	**Solvent A (%)**	**Solvent B (%)**	**Flow Rate (mL)**
0	99.5	0.5	0.35
4	30	70	0.35
4.5	2	98	0.35
5.4	2	98	0.35
5.6	99.5	0.5	0.35
11	99.5	0.5	0.35

HILIC Neg Gradient Profile			
**Time (min)**	**Solvent A (%)**	**Solvent B (%)**	**Flow Rate (mL)**
0	95	5	0.5
3.5	50	50	0.5
5.5	5	95	0.5
6.5	5	95	0.5
6.7	95	5	0.5
11	95	5	0.5

Supplementary Table 9: Metabolomics – MS details

Instrument	Q-Exactive		
Method	RP Pos	RP Neg	HILIC Neg
Source Type	HESI-II	HESI-II	HESI-II
Sheath Gas (au)	80	75	60
Auxiliary Gas (au)	12	15	20
Spray Voltage (kV)	4	2,75	3
Source Heater Temp. (°C)	400	400	380
Ion Transfer Tube Temp. (°C)	300	300	400
Normalized Collision Energy (au)	45	60	60
Stepped Normalized Collision Energy (%)	20	20	20
Mass Range (m/z)	80-1000		
MS AGC target (au)	1,00E+06		
MS Max Fill Time (ms)	60		
MS^n Ion Target (au)	2,00E+05		
MS^n Max Time (ms)	120		
MS^n Isolation Window (m/z)	3		
MS^n Dynamic Exclusion Time (s)	3		
