# Supplementary material for: High-fat diet fuels prostate cancer progression by rewiring the metabolome and amplifying the MYC program
Source: Nat Commun. 2019 Sep 25;10:4358. doi: 10.1038/s41467-019-12298-z (PMC6761092; doi:10.1038/s41467-019-12298-z)
Supplement: Supplementary file 3 — Description of Additional Supplementary Files [file 41467_2019_12298_MOESM3_ESM.pdf]

Description of Additional Supplementary Files

File Name: Supplementary Data 1
Description: Histopathologic analyses – Percent of prostatic intraepithelial neoplasia

File Name: Supplementary Data 2
Description: Metabolomics data (VP) – Scaled data and statistics

File Name: Supplementary Data 3
Description: Metabolomics data (VP) – Metabolites and pathways altered by high-fat diet in a MYC context

File Name: Supplementary Data 4
Description: MSEA (VP) – HFD_MYC vs CTD_MYC

File Name: Supplementary Data 5
Description: Metabolomics data (VP) – Metabolites and pathways altered by high-fat diet in a WT context

File Name: Supplementary Data 6
Description: Global chromatin profiling – Normalized values

File Name: Supplementary Data 7
Description: GSEA Hallmark – Murine VP

File Name: Supplementary Data 8
Description: GSEA Chemical and genetic perturbations – Murine VP

File Name: Supplementary Data 9
Description: Murine prostatic MYC signature

File Name: Supplementary Data 10
Description: GSEA Hallmark – HPFS/PHS animal fat intake

File Name: Supplementary Data 11
Description: MYC_targets_V1 – HPFS/PHS leading edge, non-leading edge and randomly picked genes

File Name: Supplementary Data 12
Description: GSEA Hallmark – HPFS/PHS saturated fat intake

File Name: Supplementary Data 13
Description: GSEA Hallmark – HPFS/PHS monounsaturated fat intake

File Name: Supplementary Data 14
Description: GSEA Hallmark – HPFS/PHS polyunsaturated fat intake

File Name: Supplementary Data 15
Description: Metabolomics data (Serum) – Scaled data and statistics

File Name: Supplementary Data 16
Description: Metabolomics data (VP) – Raw data

File Name: Supplementary Data 17
Description: Metabolomics data (VP) – OrigScale data

File Name: Supplementary Data 18
Description: Metabolomics data (Serum) – Raw data

File Name: Supplementary Data 19
Description: MSEA – Hand-curated metabolite sets

File Name: Supplementary Data 20
Description: Global chromatin profiling – Histone marks and peptides targeted

File Name: Supplementary Data 21
Description: Saturated fat intake by quintile and cohorts
