# Supplementary material for: High-fat diet fuels prostate cancer progression by rewiring the metabolome and amplifying the MYC program
Source: Nat Commun. 2019 Sep 25;10:4358. doi: 10.1038/s41467-019-12298-z (PMC6761092; doi:10.1038/s41467-019-12298-z)
Supplement: Supplementary file 25 — Reporting Summary [file 41467_2019_12298_MOESM25_ESM.pdf]

nature research

Corresponding author(s): Myles Brown, Massimo Loda

Last updated by author(s): Jul 30, 2019

Reporting Summary

Nature Research wishes to improve the reproducibility of the work that we publish. This form provides structure for consistency and transparency in reporting. For further information on Nature Research policies, see Authors & Referees and the Editorial Policy Checklist.

Statistics

For all statistical analyses, confirm that the following items are present in the figure legend, table legend, main text, or Methods section.

n/a	Confirmed	
☐	☒	The exact sample size (*n*) for each experimental group/condition, given as a discrete number and unit of measurement
☐	☒	A statement on whether measurements were taken from distinct samples or whether the same sample was measured repeatedly
☐	☒	The statistical test(s) used AND whether they are one- or two-sided<br>*Only common tests should be described solely by name; describe more complex techniques in the Methods section.*
☐	☒	A description of all covariates tested
☐	☒	A description of any assumptions or corrections, such as tests of normality and adjustment for multiple comparisons
☐	☒	A full description of the statistical parameters including central tendency (e.g. means) or other basic estimates (e.g. regression coefficient) AND variation (e.g. standard deviation) or associated estimates of uncertainty (e.g. confidence intervals)
☐	☒	For null hypothesis testing, the test statistic (e.g. *F*, *t*, *r*) with confidence intervals, effect sizes, degrees of freedom and *P* value noted<br>*Give P values as exact values whenever suitable.*
☒	☐	For Bayesian analysis, information on the choice of priors and Markov chain Monte Carlo settings
☒	☐	For hierarchical and complex designs, identification of the appropriate level for tests and full reporting of outcomes
☒	☐	Estimates of effect sizes (e.g. Cohen's *d*, Pearson's *r*), indicating how they were calculated

Our web collection on statistics for biologists contains articles on many of the points above.

Software and code

Policy information about availability of computer code

Data collection	No software was used for data collection.
Data analysis	We have spelled out all the computational analyses and software used for each experiment in detail in the online methods.

For manuscripts utilizing custom algorithms or software that are central to the research but not yet described in published literature, software must be made available to editors/reviewers. We strongly encourage code deposition in a community repository (e.g. GitHub). See the Nature Research guidelines for submitting code & software for further information.

Data

Policy information about availability of data

All manuscripts must include a data availability statement. This statement should provide the following information, where applicable:

- Accession codes, unique identifiers, or web links for publicly available datasets
- A list of figures that have associated raw data
- A description of any restrictions on data availability

Data are available from the corresponding authors upon request. Raw metabolomics data generated by Metabolon were deposited on MetaboLights and are available through the study identifier MTBLS135. Raw, scaled metabolomics data, and statistics were also provided as supplementary tables. The sequencing data reported in this paper (ChIP-seq and RNA-seq) were deposited on NCBI Gene Expression Omnibus (GEO) and are accessible through GEO Series accession number GSE90912. Human gene expression data is available through GSE62872.
Figures that have associated raw data:
Fig 1a (source data), Fig 1b (SData 1), Fig 1d (source data), Fig 1e/f/i (SData 2/16/17, MTBLS135), Fig 1g (SData 3/5), Fig 1h (SData 4);
Fig 2a/b/c (SData 6), Fig 2d (ChIP-seq: GSE90912), Fig 2e (RNA-seq: GSE90912);
Fig 3a (SData 7, RNA-seq: GSE90912), Fig 3b (SData 9, RNA-seq: GSE90912), Fig 3c/d/e (ChIP-seq GSE90912);
Fig 4a/b (SData 10/12/13/14, Human gene expression GSE62872), Fig 4e (RNA seq: GSE90912);
Fig S1a (source data), Fig S1d/e (SData 15/18, MTBLS135), Fig S1g/h/i (SData 2/16/17, MTBLS135);

Fig S2a/b (SData 6), Fig S2c (RNA-seq: GSE90912);
Fig S3a (source data), Fig S3b (SData 8, RNA-seq: GSE90912).

Field-specific reporting

Please select the one below that is the best fit for your research. If you are not sure, read the appropriate sections before making your selection.

☒ Life sciences ☐ Behavioural & social sciences ☐ Ecological, evolutionary & environmental sciences

For a reference copy of the document with all sections, see nature.com/documents/nr-reporting-summary-flat.pdf

Life sciences study design

All studies must disclose on these points even when the disclosure is negative.

Sample size	Sample size for metabolomics and histological evaluation was estimated empirically based on previous literature data, using the same model [Priolo et al., Cancer Research 2014; Kobayashi et al., Cancer Research 2008] for sufficient statistical power. Sample size for RNA-seq was estimated empirically for sufficient statistical power based on literature. For Ki-67 analysis, we performed sample size calculation using the software G*power version 3.1, extrapolating the effect size (d=around 0.87) from the data of Kobayashi et al. [Kobayashi et al., Cancer Research 2008] in MYC mice fed with a high-fat diet. Based on this assumption, we calculated that at least 22 mice/group should be used to detect a significant difference in Ki-67 positivity using a two-sided t-test for change in mean between two independent groups, with an alpha-error of 0.05 and a priori power of 0.8.
Data exclusions	In the entire study, no data were excluded except for the analysis of insulin levels measured by ELISA. In that analysis, 3 samples were excluded, 2 were identified as outliers (outlier identification done using the ROUT method, Q=0.1%) and one was under the detection limit of the assay.
Replication	The murine analyses reported comparing genotype / diet conditions uses a minimum of 3 independent prostate lobes (RNA seq, global chromatin profiling), 6 (metabolomics), between 16 to 24 (immunohistochemistry and histopathology) or two pools of biological replicates comprised of at least 8 samples each (ChIP-seq) harvested from distinct mice. Number of independent samples is clearly stated in the manuscript.
Randomization	For preclinical studies, litters were randomly assigned to one of the two purified diets.
Blinding	For preclinical studies, group allocation to diet regimen was performed in a non-blinded fashion. Histopatholy and immunohistochemistry analyses were performed by three expert uropathologists who were blind to the experimental conditions.

Reporting for specific materials, systems and methods

We require information from authors about some types of materials, experimental systems and methods used in many studies. Here, indicate whether each material, system or method listed is relevant to your study. If you are not sure if a list item applies to your research, read the appropriate section before selecting a response.

Materials & experimental systems

n/a	Involved in the study	
☐	☒	Antibodies
☐	☒	Eukaryotic cell lines
☒	☐	Palaeontology
☐	☒	Animals and other organisms
☐	☒	Human research participants
☒	☐	Clinical data

Methods

n/a	Involved in the study	
☐	☒	ChIP-seq
☒	☐	Flow cytometry
☒	☐	MRI-based neuroimaging

Antibodies

Antibodies used	We have used only commercially available antibodies.<br>List of antibodies used:<br>1) anti-Ki-67 antibody (#VP-RM04 (clone SP6), Vectors Laboratories);<br>2) anti-H4K20me1 antibody (#ab9051, Abcam);<br>3) anti-PHF8 (#A301-772A, Bethyl Laboratories);<br>4) rabbit monoclonal [Y69] anti-c-MYC (#ab32072, Abcam);<br>5) rabbit polyclonal anti-Beta-Actin (#4967, Cell Signaling Technology).
Validation	All the antibodies are widely published and have quality control tested per the company's standard procedure.<br>For immunohistochemistry, conditions were validated using the positive controls indicated by the manufacturer. For western blot, lysates from MyC-CaP cells harboring the MYC transgene was included as a positive control.<br>1) anti-Ki-67 antibody (#VP-RM04 (clone SP6), Vectors Laboratories); 46 references were cited using this antibody on the

company datasheet (https://www.labome.com/product/Vector-Laboratories/VP-RM04.html);
2) anti-H4K20me1 antibody (#ab9051, Abcam); 85 references were cited using this antibody on the company's datasheet (https://www.abcam.com/histone-h4-mono-methyl-k20-antibody-chip-grade-ab9051-references.html#active-tab);
3) anti-PHF8 (#A301-772A, Bethyl Laboratories); 5 references were cited using this antibody on the company's datasheet (https://www.citeab.com/antibodies/656273-a301-772a-phf8-antibody?utm_campaign=Widget+Al+Citations&utm_medium=Widget&utm_source=Bethyl+Laboratories&utm_term=Bethyl+Laboratories);
4) rabbit monoclonal [Y69] anti-c-MYC (#ab32072, Abcam); 375 references were cited using this antibody on the company's datasheet (https://www.abcam.com/c-myc-antibody-y69-ab32072.html);
5) rabbit polyclonal anti-Beta-Actin (#4967, Cell Signaling Technology); 1278 references were cited using this antibody on the company's datasheet (https://www.cellsignal.com/products/primary-antibodies/b-actin-antibody/4967?N=4294960387&fromPage=plp).

Eukaryotic cell lines

Policy information about cell lines

Cell line source(s)	Murine MYC-CaP cells were kindly provided by Dr. Charles Sawyers, Memorial Sloan Kettering Cancer Center, New York, NY, US.
Authentication	Cells were authenticated via STR profiling (DDC Medical, January 16th 2015).
Mycoplasma contamination	Cells were tested negative for mycoplasma contamination using MycoAlert™ Mycoplasma Detection Kit (Lonza). Experiments were performed with mycoplasma negative cells.
Commonly misidentified lines (See ICLAC register)	No commonly misidentified cell lines were used.

Animals and other organisms

Policy information about studies involving animals; ARRIVE guidelines recommended for reporting animal research

Laboratory animals	FVB Hi-MYC mice (strain number 01XK8), expressing the human c-MYC transgene in prostatic epithelium (heterogyzogous for MYC transgene), were obtained from the National Cancer Institute Mouse Repository at Frederick National Laboratory for Cancer Research. Hi-MYC mice were bred with wild-type FVB mice to obtain male wild-type and Hi-MYC mice to be used for this research. For the experimental groups, upon weaning (3 weeks), Hi-MYC and wild type littermates (WT), were fed a purified control diet (CTD; Harlan Laboratories, TD.130838) consisting of 10% fat, or a high-fat diet (HFD; Harlan Laboratories, TD. 06414) consisting of 60% fat until 12, 24 or 36 weeks of age. For dietary intervention experiments, mice assigned an HFD were switched to a CTD at 10 weeks of age for the folllowing 2 weeks until the experimental endpoint. Breeding pairs were kept under chow diet. Animals were kept on a 12-hour light / 12-hour dark cycle, with controlled temperature and humidity, and allowed free access to food and water at the Dana-Farber Cancer Institute Animal Resources Facility. Food was changed on a weekly basis, and mice were weighed every three weeks, starting at weaning.
Wild animals	NA
Field-collected samples	NA
Ethics oversight	The study was performed under the protocol 13-049. The animal protocol was reviewed and approved by the Dana-Farber Cancer Institute Institutional Animal Care and Use Committee (IACUC), and was in accordance with the Animal Welfare Act.

Note that full information on the approval of the study protocol must also be provided in the manuscript.

Human research participants

Policy information about studies involving human research participants

Population characteristics

Two prospective studies were used in our study, namely the Physicians' Health Study (PHS) and the Health Professionals Follow-up Study (HPFS). Characteristics of the cases analyzed in the study are detailed in Table 1 of the manuscript.

In both studies, participants were followed by means of regular questionnaires, and self-reported data on diet, lifestyle behaviours, medical history, and disease outcomes were collected. The incidence of prostate cancer cases in this population was confirmed by reviewing medical records and pathology reports. Following the confirmation of diagnosis, archival formalin-fixed paraffin-embedded (FFPE) of prostate tissue specimens , collected during radical prostatectomy or transurethral resection of the prostate, were retrieved.

Pathologists undertook a standardized histopathologic review, including Gleason grading (Ref. 72 in the manuscript), and standardized clinical data were abstracted from medical records. Deaths were ascertained via mail, telephone, and through periodic systematic searches of the National Death Index. Lethal prostate cancer was defined as the occurrence of distant metastases, or death due to prostate cancer. Men were followed through March 2011 for PHS and through December 2011 for HPFS. We obtained written informed consent from all participants, and the study was approved by institutional review boards at the Harvard T.H. Chan School of Public Health and Partners Health Care.

As validation cohorts, we utilized genome-wide expression profiles of 751 patients with metastatic outcome follow-up from the Decipher Genomic Resource Information Database (GRID; NCT02609269).

Recruitment	PHS I and II began in 1982 and 1997, respectively, as randomized trials of aspirin (PHS I) and dietary supplements (PHS II), and enrolled 29,067 male U.S. physicians for the primary prevention of cardiovascular disease and cancer (Ref. 67, 68, 69, 70 in the manuscript). The HPFS was initiated in 1986, when 51,529 U.S. men, 40-75 years of age and working in health professions, completed a biennial questionnaire mailed to them (Ref. 71 in the manuscript).<br><br>As validation cohorts, we utilized genome-wide expression profiles of 751 patients with metastatic outcome follow-up from the Decipher Genomic Resource Information Database (GRID; NCT02609269). These patients were pooled from four studies of either case-cohort or cohort design. Patients for these studies came from four institutes: Thomas Jefferson University (TJU; n = 139; Ref. 79 in the manuscript), Johns Hopkins Medical Institutions-I (JHMI-I; n = 260; Ref. 80 in the manuscript), Mayo Clinic (n = 235; Ref. 81 in the manuscript), Cedars-Sinai (n = 117; Ref. 82 in the manuscript).
Ethics oversight	Written informed consent was obtained from all participants (PHS/HPFS), and the study was approved by institutional review boards at the Harvard T.H. Chan School of Public Health and Partners Health Care.

Note that full information on the approval of the study protocol must also be provided in the manuscript.

ChIP-seq

Data deposition

☒ Confirm that both raw and final processed data have been deposited in a public database such as GEO.

☐ Confirm that you have deposited or provided access to graph files (e.g. BED files) for the called peaks.

Data access links<br>*May remain private before publication.*	https://www.ncbi.nlm.nih.gov/geo/query/acc.cgi?acc=GSE90912
Files in database submission	Merge_Pool_1_2_CTD_WT_PHF8.fq.gz<br>Merge_Pool_3_4_CTD_Het_PHF8.fq.gz<br>Merge_Pool_5_6_HFD_WT_PHF8.fq.gz<br>Merge_Pool_7_8_HFD_Het_PHF8.fq.gz<br>Merge_Pool_1_2_CTD_WT_H4K20me1.fq.gz<br>Merge_Pool_3_4_CTD_Het_H4K20me1.fq.gz<br>Merge_Pool_5_6_HFD_WT_H4K20me1.fq.gz<br>Merge_Pool_7_8_HFD_Het_H4K20me1.fq.gz<br>Merge_Pool_1_2_CTD_WT_PHF8.bigwig<br>Merge_Pool_3_4_CTD_Het_PHF8.bigwig<br>Merge_Pool_5_6_HFD_WT_PHF8.bigwig<br>Merge_Pool_7_8_HFD_Het_PHF8.bigwig<br>Merge_Pool_1_2_CTD_WT_H4K20me1.bigwig<br>Merge_Pool_3_4_CTD_Het_H4K20me1.bigwig<br>Merge_Pool_5_6_HFD_WT_H4K20me1.bigwig<br>Merge_Pool_7_8_HFD_Het_H4K20me1.bigwig
Genome browser session<br>(e.g. UCSC)	No session was generated.

Methodology

Replicates	Merged two pools of biological replicates each comprised of multiple samples harvested from distinct 12-week-old mice:<br>CTD_WT Pool #1 (12 mice) and Pool #2 (13 mice);<br>CTD_MYC Pool #3 (8 mice) and Pool #4 (8 mice);<br>HFD_WT Pool #5 (14 mice) and Pool #6 (15 mice);<br>HFD_MYC Pool #7 (8 mice) and Pool #8 (8 mice).
Sequencing depth	Sequencing was done at 75bp, single-ended.
Antibodies	1) anti-H4K20me1 antibody (#ab9051, Abcam, lot: GR175668-2);<br>2) anti-PHF8 (#A301-772A, Bethyl Laboratories, lot: A301-772A-3).
Peak calling parameters	Peak centric analyses were not performed.
Data quality	Quality of ChIP-seq experiments was determined by mapping signal at known promoter and transcription factor binding sites. High signal above background at expected sites of enrichment indicated successful enrichment for the protein of interest. In addition, active genomic loci were cross-referenced with RNA-seq to determine active transcription.
Software	bowtie2 2.2.1<br>cutadapt 1.16<br>FastQC v0.11.5<br>samtools 1.9<br>bedtools v2.27.1
